# Implementation intentions as an acceptable health behaviour change strategy? Insights from people with lower socio‐economic position in think‐aloud interviews

**DOI:** 10.1111/bjhp.70090

**Published:** 2026-07-10

**Authors:** Loes van den Bekerom, Laurens C. van Gestel, Satu Koivusaari, Jet Bussemaker, Marieke A. Adriaanse

**Affiliations:** ^1^ Health Campus the Hague/Department of Public Health and Primary Care Leiden University Medical Center The Hague/Leiden the Netherlands; ^2^ Health, Medical and Neuropsychology Unit Leiden University Leiden the Netherlands; ^3^ The Institute of Public Administration, Leiden University Leiden the Netherlands

**Keywords:** acceptability, feasibility, health behaviour change, implementation intentions, qualitative, socio‐economic position (SEP), think aloud

## Abstract

**Objectives:**

Implementation intentions are simple action plans that may help people with lower socio‐economic position (SEP) to navigate their behaviour in environments that undermine their healthy intentions. Implementation intentions hold potential in being a relatively simple, but remarkably effective behaviour change technique. However, it remains unclear whether such a micro‐intervention fits the daily realities, needs and skills of lower SEP groups. We aimed to explore how adults with lower SEP (1) perceive (generating) implementation intentions for changing their health behaviour, and (2) independently generate implementation intentions.

**Methods:**

Adults living in lower SEP neighbourhoods (*n* = 15) participated in semi‐structured interviews. Participants were instructed to think aloud while independently generating an implementation intention following written instructions. Interview data were thematically analysed, and the plans were evaluated.

**Results:**

Participants perceived health as a central value in life, which translated into high readiness for health behaviour change. Implementation intentions were perceived as helpful and motivating because they provide structure, support small, doable steps and stimulate conscious decisions and reflections. Participants expressed strong motivation to use implementation intentions in daily life. However, although participants expressed that generating their plan was pleasant and easy, guidance was frequently requested and provided during plan formation. Besides, generated plans often lacked the essential if–then structure and concreteness.

**Conclusions:**

Implementation intentions appear acceptable and appealing as a behaviour change strategy for adults with lower SEP. However, independent formation of well‐structured, actionable plans is challenging. Guided support is needed to ensure successful use of implementation intentions in lower SEP populations.

**Statement of Contribution**

**What is already known on this subject?**

- Adults with lower socio‐economic position (SEP) are highly motivated to live healthily but face substantial barriers to translate healthy intentions into action.
- Implementation intentions are simple if–then plans that have strong effects in the general population, but the evidence in lower SEP groups is limited and mixed.
- There is limited insight into whether using simple implementation intentions for health behaviour change fits the complex realities, needs and skills of lower SEP populations.

**What does this study add?**

- Adults with lower SEP view implementation intentions as helpful and motivating, and highly intend to use them.
- Independent plan formation is challenging and plans often lack if–then structure and concreteness.
- Guided support is crucial for generating actionable implementation intentions in lower SEP groups.

*‘Strong effects of simple plans’ (*Gollwitzer, 1999, *p. 493) … But what if the problems are so complex?*

## INTRODUCTION

People with lower socio‐economic position (SEP), i.e., one's relative standing in a societal hierarchy typically determined by income, education and occupation, as well as subjective perceptions of social status and class (APA, 2022), are at greater risk of developing preventable chronic diseases (Adler & Stewart, 2010). This is for a large part due to higher prevalence of unhealthy behaviour such as smoking, physical inactivity and poorer dietary patterns (Stringhini et al., 2017). Importantly, research suggests that individuals with lower SEP are often highly motivated to act healthily, but face harsh circumstances on a daily basis that impede their ability to act upon their healthy intentions (Koivusaari et al., 2025; McCoy et al., 2024). Consider, for example, a recently divorced father of three young children who combines caregiving responsibilities with physically demanding shift‐based construction work, while coping with financial strain, chronic stress, limited sleep and health problems. Although he might be motivated to adopt healthier habits, the realities of his daily life may make it appealing to smoke to momentarily ease stress and to order take‐away meals from the fast‐food shops around the corner. Such daily realities illustrate the broader challenge of translating healthy intentions into action within socio‐economically disadvantaged circumstances.

The physical and social environments of individuals with lower SEP strongly shape their health behaviours, often in ways that constrain rather than support healthy choices. That is, lower SEP neighbourhoods are more likely to contain higher densities of fast‐food outlets, convenience stores, tobacco marketing and fewer opportunities for (safe) physical activity (e.g., Cummins & Macintyre, 2006; Fleischhacker et al., 2011; Lee et al., 2015; Wilson et al., 2004). In such environments, there is frequent exposure to temptation and healthier alternatives are logistically or financially harder to access (Darmon & Drewnowski, 2015). Structurally targeting these environments may be the most effective way to reduce socio‐economic health inequalities (Gepkens & Gunning‐Schepers, 1996). However, large‐scale systematic changes are complex, often dependent on political will and are therefore slow. This leaves individuals with lower SEP to deal with daily contexts that frequently undermine healthy choices and puts an emphasis on their motivation in such unsupportive environments.

This implies that having healthy intentions alone is not sufficient to ensure behavioural enactment. Intentions explain only a limited proportion of actual behaviour (Sheeran et al., 2005). This was already recognized in classical behaviour change models, acknowledging that health behaviour is not only shaped by intentions but also by perceived behavioural control, i.e., the extent to which individuals feel control over performing a behaviour (e.g., the Theory of Planned Behaviour: Ajzen, 1991). However, beyond intentions and beliefs about behavioural control, successful behaviour change also depends on actual action control, i.e., the ability to initiate, monitor and maintain goal‐directed behaviour when confronted with competing demands, distractions or (environmental) barriers (e.g., Gollwitzer, 1999; Gollwitzer & Sheeran, 2006; Schwarzer, 2008). This ability is particularly relevant in light of the contexts individuals with lower SEP face, where competing demands, chronic stress, limited resources and unsupportive environments may undermine the enactment of even strongly held intentions. Thus, while motivation to live healthily is high in lower SEP groups (Koivusaari et al., 2025), immediate tools are needed to help translate such intentions into action within challenging surroundings (Godin et al., 2010; McCoy et al., 2024). It raises the question which behaviour change strategies are acceptable, feasible and effective to help people with lower SEP to navigate their behaviour in discouraging environments.

### Strong effects of simple plans?

One of the most prominent behaviour change strategies supporting people in translating their healthy intentions in healthy behaviour in unsupportive environments, is the formation of implementation intentions. Implementation intentions are specific if–then plans that specify when, where and how to act on a given goal (Gollwitzer, 1999). They link anticipated critical situations (‘If situation *x* arises’) to goal‐directed responses (‘then I will initiate goal‐directed response *y*’; Gollwitzer, 1999). Implementation intentions can effectively make the mental representation of the critical situation highly accessible and create a strong associative link between this mental representation and the goal‐directed response (Wieber et al., 2015). By prespecifying when, where and how to act, implementation intentions are thought to facilitate automatic initiation of behaviour in a specific context and to reduce reliance on effortful, conscious self‐control processes, thereby supporting action control at the moment of opportunity (Gilbert et al., 2009; Gollwitzer & Sheeran, 2006). Their effectiveness is best summarized by the title of Gollwitzer's original paper ‘*strong effects of simple plans’* (1999, p. 493). It describes how this strategy is relatively simple to use, but—especially given its simplicity—highly effective in bridging the intention‐behaviour gap in the general population across numerous health behaviours (Adriaanse et al., 2011; Gollwitzer & Sheeran, 2006; Silva et al., 2018).

Using implementation intentions may be particularly relevant for individuals with lower SEP, who are highly motivated to live a healthy live (e.g., Koivusaari et al., 2025), but whose cognitive and material resources that can support this may be limited due to the conditions they live in. That is, people with lower SEP often experience harsh circumstances, chronic stress or poverty which can tax cognitive abilities that are needed for decision making, problem solving and attentional capacity (e.g., Adler & Rehkopf, 2008; Haushofer & Fehr, 2014; Kraft & Kraft, 2021; Mani et al., 2013; Sheehy‐Skeffington, 2019; Van der Veer et al., 2024). They also appear to experience more difficulty with engaging in self‐regulation (Moffitt et al., 2011) and planning (Bieleke & Keller, 2021; Prenda & Lachman, 2001) in general. This all may hinder one's control over behaviour and the translation of healthy intentions in actual behaviours, and it is exactly in these contexts that implementation intentions are found to be most effective (Webb & Sheeran, 2006).

Although in theory lower SEP populations may thus strongly benefit from this strategy, empirical evidence in this population is scarce and demonstrates mixed effects. While some studies indicate positive effects among people with lower SEP or related variables (e.g., higher perceived stress or lower health literacy; Abbott et al., 2020; Armitage & Arden, 2010; Armitage & Sprigg, 2010; Ayre et al., 2019; Voigt et al., 2022), others do not find such effects (DeBiasse et al., 2017; Voigt et al., 2022).

Yet, these findings are difficult to interpret without understanding whether implementation intentions are actually relevant, acceptable and feasible for people with lower SEP. Evaluating effectiveness alone provides limited insights if individuals do not perceive the strategy as useful, are unwilling to use it, or struggle to apply it correctly in daily life. Only if the strategy aligns with the realities of their daily lives, it makes sense to evaluate its potential for sustained adoption and effectiveness in practice. Therefore, understanding whether implementation intentions fit the context, needs and skills of individuals with lower SEP is a crucial first step.

### … But what if the problems are so complex?

It can be questioned whether strategies such as implementation intentions are truly appealing or feasible when the daily challenges people face are substantial. Does ‘strong effects of simple plans’ apply when individuals' problems are so complex? For people in lower socio‐economic contexts like the single father described earlier, a ‘simple plan’ may feel far from simple and impossible to execute in the face of daily challenges. Besides, the focus of implementation intentions on one specific behaviour change (e.g., taking a walk after work or choosing a piece of fruit as a snack) may feel irrelevant or trivial for populations frequently experiencing relatively large health problems. Although theoretically, the simplicity of the strategy is what makes this strategy particularly promising (Gollwitzer, 1999), it may also reduce its motivational appeal for eliciting behaviour change among individuals handling multiple priorities. Beyond motivation to change the health behaviour specified in the plan (i.e., goal commitment: Gollwitzer & Sheeran, 2006), commitment to using the plan itself is essential for its effectiveness (Gollwitzer & Oettingen, 2016).

To date, it remains unclear whether individuals with lower SEP consider implementation intentions as a suitable and feasible strategy in their daily lives and whether they are motivated to use this strategy. Strikingly, only one study quantitatively examined acceptability (DeBiasse et al., 2017), demonstrating that individuals with lower SEP generally find generating implementation intentions enjoyable, easy and motivating in terms of making positive changes in their health behaviour. Despite these promising initial findings, evidence on how well implementation intentions fit with the needs, interests and daily realities of lower SEP populations remains scarce. This underscores the need to explore how individuals with lower SEP perceive using implementation intentions for health behaviour change.

Effective use of implementation intentions also depends on how the plans are formulated. Poorly specified or vague implementation intentions may fail to support behaviour change (Gollwitzer & Sheeran, 2006). Formulating effective implementation intentions requires identifying a recognizable and frequently occurring cue, identifying a goal‐directed behavioural response that can be implemented and linking them in a specific if–then format (Gollwitzer et al., 2010). Still, it can be difficult (and thus not so ‘simple’) to identify and specify the critical cue because individuals have poor introspection into the triggers of habitual behaviour (Adriaanse et al., 2011; Adriaanse & Verhoeven, 2018). Yet, no studies examined how individuals with lower SEP independently generate implementation intentions. Many current studies among individuals with lower SEP provided additional guidance to formulate their implementation intention (e.g., a volitional help sheet; Armitage & Arden, 2010, a prescribed implementation intention; Armitage & Sprigg, 2010). However, such guidance may steer individuals with lower SEP towards goals, situations and behaviours that are less personally relevant or feasible within their daily life context. Generating concrete implementation intentions that are personally relevant and feasible is essential for its effectiveness, but this demands cognitive capacities. It is therefore yet to be examined how adults with lower SEP independently generate implementation intentions.

### The present study

The present qualitative study aims to explore how adults with lower SEP (1) perceive (generating) implementation intentions to change their health behaviour, and (2) independently generate implementation intentions. The study employed semi‐structured interviews, in which a think‐aloud methodology was applied when participants generated implementation intentions. The semi‐structured interviews offer an understanding of participants' perceptions of their formulated plan, the usefulness of the technique and its fit with their needs, preferences and daily life. The think‐aloud approach yields unique insights into participants' thoughts, facilitators and challenges during plan formation. This combined approach allows to assess both the acceptability of the technique and the feasibility and structure of self‐generated implementation intentions in a lower SEP population.

## MATERIALS AND METHODS

### Design and philosophical approach

A qualitative approach was used, where a think‐aloud method (Ericsson & Simon, 1993; Willis, 2004) was applied during a semi‐structured interview. This approach was based on a qualitative study on implementation intentions performed among older adults (Bösch et al., 2023). The current study was designed as an applied, pragmatic qualitative study (Creswell, 2007), commonly used in applied health research and implementation science, with a focus on generating actionable insights to inform intervention design and potential application of implementation intentions in a lower SEP context. Consistent with this approach, we also evaluated the implementation intentions that participants had formulated.

### Participants

Eligibility criteria included being (a) an adult living in neighbourhoods in The Hague, the Netherlands characterized by lower SEP (based on SES‐WOA scores; Statistics Netherlands, 2023), (b) proficient in the Dutch language and (c) able to independently participate in the study. We aimed to recruit participants until data saturation would be reached. This was reached at 15 participants, since no new properties, dimensions and themes emerged from the data of this additional interview.

### Procedure

#### Recruitment and consent

A convenience sample was recruited from local community centres and food aid stores in two lower SEP neighbourhoods and through snowball sampling via participants' networks. It was monitored whether included participants had various ages, genders and ethnicities. During recruitment, the study was explained and potential participants received an information letter to read at their own pace. They were contacted at a later moment, could ask questions and received further information if needed. Interviews were scheduled at a quiet location preferred by the participants (e.g., at home, the community centre or the university). During the interview appointment, the study was explained again, remaining questions were addressed and informed consent was obtained.

#### Interviews

**FIGURE 1:**
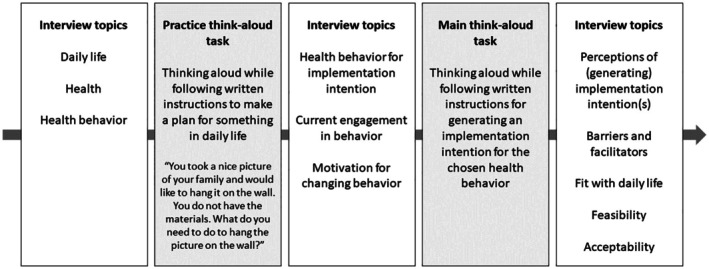
Procedure of semi‐structured interviews.

The semi‐structured interview was audio‐recorded and began with an introductory question to build rapport. It continued with questions about participants' daily life, (their) health and health behaviour (see Figure 1 for an overview of the semi‐structured interview and Data S1 for the Dutch and English (translated) interview guide, including the think aloud instructions). Next, participants performed an unrelated practice think‐aloud task to get them acquainted with thinking aloud while planning something following written instructions. They were instructed to read the instructions aloud and verbalize all their thoughts during the task, including how they understood the instructions, what they thought about them and about their own answers (i.e., level 1 and level 2 think aloud instructions: Ericsson & Simon, 1993; Van Someren et al., 1994). After the practice task, participants chose a health behaviour for their plan. Participants were free to select any behaviour that they aimed to change and perceived to be related to their health. Follow‐up questions included their current engagement in that behaviour and motivation for changing it. Participants then completed the main think‐aloud task, by thinking aloud while independently generating an implementation intention for the chosen health behaviour following written step‐by‐step instructions in simple Dutch language (for the practice and main task, in Dutch, see Data S2). After the task, participants were asked about their perceptions of (generating) implementation intentions, barriers and facilitators for generating them using the instructions and the feasibility, acceptability and fit with daily life of the generated plan and implementation intentions in general. At the end of the appointment, participants completed a short demographic questionnaire, were thanked for their participation and received a 20 euro voucher.

All interviews took on average 1 h (range 45–90 min).^1^ Data were collected from mid‐December 2024 to early June 2025 (excluding 4 weeks around Christmas, since this period and new year's resolutions could influence the findings). The instructions of the main think‐aloud task and the interview guide were continuously evaluated and updated when deemed necessary.

#### Ethics

The department's science committee positively assessed the current study's quality and feasibility (#WSC‐2024‐41/SP) and the Medical Ethics Review Board of Leiden University Medical Center confirmed that the Medical Research Involving Human Subjects Act (WMO in Dutch) does not apply to the current study according to Dutch standards (#24–3077).

### Data analysis

Data collection and analysis were performed iteratively. All interviews, including the think aloud data, were transcribed verbatim by one researcher (LvdB) and were thematically analysed in ATLAS.ti v24.2.0 (ATLAS.ti Scientific Software Development GmbH, 2024) using open coding and a general inductive approach (Braun & Clarke, 2012; Thomas, 2006). No a priori framework or model was imposed onto the data, but the study design and related analysis were guided by theories about implementation intentions (e.g., Gollwitzer, 1999) on essential aspects of successful implementation intention formulation and effectiveness (e.g., goal commitment as a prerequisite, plan commitment, the specificity of the plan and the if–then structure including a personally relevant cue and goal‐directed response) and by acceptability and feasibility research (e.g., perceived usefulness, perceived effectiveness, motivation and intention to use implementation intentions, perceived feasibility and independent plan formation).

The first author (LvdB) read all the transcripts to become familiar with the data and made a list of initial topics and codes to guide the analysis, while remaining open to inductive coding. Two researchers (LvdB and SK) independently coded the first four interviews and compared the codes and topics after each interview by going through the codes and quotations of each interview from its beginning to the end to reach consensus. Inconsistencies were discussed with a third researcher (LvG) and used to refine code definitions, reduce overlap between them and add new codes. This guaranteed the quality of data analysis and informed minor refinements to the interview materials to improve clarity and reduce ambiguity (e.g., rephrasing the question about the role of health in one's life, emphasizing that the instructions should be read ‘out loud’ and simplifying the implementation intention instructions by using only one example if–then plan). After the fourth interview, no inconsistencies between the two coders existed, resulting in a code framework used for coding the remaining interviews while allowing for continued inductive refinement. The remaining interviews were coded by one researcher (LvdB). This resulted in 24 main codes with numerous subcodes (i.e., 672 unique codes in total). For example, the main code ‘Attitude about action plan’ included subcodes such as ‘Perceived effectiveness’, ‘Support from plan’ and ‘No support from plan’. LvdB then combined codes and recurring topics into overarching themes. For example, codes related to the main codes of ‘Attitude about action plan’ and ‘Attitude about strategy’ were combined into the theme ‘Implementation intentions are perceived as helpful for changing health behaviour’. Themes and their interrelations were continuously discussed and refined within the research team.

### Qualitative rigour

Reflexivity was embedded throughout the research process and is described in Data S3. In addition, several strategies were used to strengthen the qualitative rigour of the study, such as investigator triangulation, data source triangulation and methodological triangulation (e.g., Carter, 2014; Donkoh & Mensah, 2023). Specifically, two researchers coded and discussed (eventually with a third researcher in case of discrepancies) the first four interviews until consensus was reached. Themes and interpretations were regularly discussed within the interdisciplinary research team. Data source triangulation involved including participants with diverse demographic backgrounds from different lower SEP neighbourhoods. For methodological triangulation, the combination of semi‐structured interviews, think‐aloud procedures and field notes further strengthened the credibility and contextual interpretation of the findings. Finally, findings were shared orally with local stakeholders and digitally via a simplified (B1‐level) fact sheet with participants for input, but that did not yield any substantive comments considering the findings.

## RESULTS

### Participants

Participants were 15 adults (9 female, 6 male; M_age_ = 47.93 SD_age_ = 15.91, range_age_ = 20–67) living in lower SEP neighbourhoods (e.g., Moerwijk, Laakkwartier and Vrederust) in The Hague, which is one of the biggest yet highly segregated cities in the Netherlands. Most participants had the Dutch ethnicity (*n* = 8, 53%), followed by or combined with (in case of multiple ethnicities) Surinamese (*n* = 4, 26.7%), Moroccan (*n* = 3, 20%), Hindustani (*n* = 2, 13.3%) and Afghan (*n* = 1, 6.7%). Most participants had a lower educational level (*n* = 7, 46.7%), followed by medium (*n* = 5, 33.3%) and higher (*n* = 3, 20%) educational levels. Educational level was categorized according to the definitions of Statistics Netherlands (2025) into lower (i.e., lower secondary education or lower vocational education at highest), medium (i.e., upper secondary education or intermediate vocational education at highest) and higher (i.e., higher professional education or university education). Despite different educational backgrounds, all participants met the inclusion criterium of living in lower SEP situations (e.g., receiving social assistance benefit or food aid).

### Main results

The main results are structured according to the two study aims. For each aim, several themes and subthemes were identified (please see Table 1).

**TABLE 1:** Identified themes and subthemes for the two study aims.

Study aim	Theme	Subtheme
Perceptions of health (behaviour change), using implementation intentions and generating implementation intentions (First aim)	Health is a central value	
	High motivation for behaviour change	
	Positive perceptions of using implementation intentions	Implementation intentions are perceived as helpful for changing health behaviour
		Intention and motivation to use implementation intentions is high
	Positive perceptions of generating implementation intentions	Generating implementation intentions is perceived as easy
		Generating implementation intentions is pleasant and helps to reflect
	Social support is an additional need	
Independently generating implementation intentions: Process and evaluation of formulated plans (Second aim)	Guidance was often requested or provided during generating implementation intentions	
	Formulated plans lack important elements	Plans are not formulated in the correct if–then structure
		Plans are not concrete

#### Perceptions of health (behaviour change), using implementation intentions and generating implementation intentions (first aim)

The results for the first aim begin with participants' perceptions of health (behaviour change), as motivation to attain the health goal is a prerequisite for effective use of implementation intentions (Gollwitzer & Sheeran, 2006). Participants strongly valued health, resulting in high readiness for behaviour change. Furthermore, they had positive perceptions of using implementation intentions, which were perceived as helpful for changing their behaviour. Participants perceived generating implementation intentions as easy and pleasant, but also mentioned to appreciate social support. These themes are further described below.

##### Health is a central value

A key theme identified was that health was considered very important, or even one of the most important aspects of participants' lives. Health was often described as the foundation that enables them to do different things in life. Facing poor health in the past or present reinforced the importance of one's health. Besides being physically healthy, feeling mentally healthy was perceived as a central component of health. This is summarized in the following statement: ‘*Health plays an important role, because without health you can't move […] Health is your machine in your life. […] Without good mental health, you can't make good choices either, for example, you can't build healthy contacts. You might get stuck. […] You won't see any opportunities. No hope sometimes. Yes, so health […] it's the pivot of our lives*.’ (Man, 51, high education).

##### High motivation for behaviour change

Reflecting the strong importance of health, participants were highly motivated to improve their health through behaviour change in daily life. Frequently mentioned daily activities to improve health included physical activity (e.g., walking through the neighbourhood, fitness or cycling) and healthy eating (e.g., consuming fruit and vegetables and less unhealthy snacks). This strong motivation was also expressed for the behaviours participants selected for their plans. While most participants aimed to implement new behaviours, some already pursued their goal but sought to improve or maintain progress. Most participants chose typical health behaviours, such as healthy eating, physical activity, better sleeping and taking more rest. Others selected outcomes that they personally linked to their health and well‐being, but that are not typical health behaviours (e.g., doing household chores or finding a buddy for social activities) or concrete behaviours at all (e.g., feeling more confident). Generally, participants expressed dissatisfaction with their current engagement in the chosen (health) behaviour and perceived changing it as necessary and beneficial for their health and well‐being. Overall, valuing health strongly translated into readiness and motivation for behaviour change.

##### Positive perceptions of using implementation intentions

###### Implementation intentions are perceived as helpful for changing health behaviour

A key theme comprised participants' positive attitudes towards the perceived effectiveness of implementation intentions and several aspects emerged of how such plans could support them in changing behaviour. Firstly, implementation intentions were generally perceived as a kind of personal rule or appointment that could help to improve behaviour (*‘To have stability, fixed times, fixed rules so to say’*, woman, 64, low education; ‘*Sort of a reminder, because when you have so much to do in daily life […] you sometimes get distracted, […] but then this helps to have it in order’*, man, 41, medium education). Besides, generating and writing down a plan was generally perceived to support conscious decision‐making about how and when to implement behaviour (e.g., ‘*think consciously about how to do it instead of just doing something’*, man, 47, high education) and getting the plan activated in one's consciousness (e.g., *‘you are mentally occupied with it, and if you are occupied with it mentally, you will automatically do the same in your body’* woman, 20, medium education). This increased the feeling that one really wants to commit to it (e.g., ‘*When I write it down, it hits me really close, like, I've made a plan. If I don't stick to it, I don't feel good’*, man, 41, low education). Finally, participants valued that implementation intentions help to start small and work on improving one's behaviour step by step. This indicates that participants generally understood the purpose of implementation intentions in terms of ‘strong effects of simple plans’ (*‘I think it is a habit that you have to learn. […] Stepping up a bit if you're already motivated’*, woman, 62, low education). Although some participants mentioned that the helpfulness of the plan could be limited (e.g., moments that are out of one's control or when experiencing pitfalls), most participants stated that there was nothing the plan could not help with. Overall, implementation intentions were perceived as helpful, specifically because they stimulate structure, consciousness, motivation and taking small steps.

###### Intention and motivation to use implementation intentions is high

Another theme was the strong willingness to use implementation intentions in daily life. Both participants' intention and motivation to implement their formulated plan were very high, as illustrated by the following statement: *‘That's my goal and what I want, and that's my plan. […] I'm going to work really hard to actually make that happen.’* (woman, 42, low education). This willingness remained high, even when participants anticipated difficulties in maintaining the behaviour, questioned feasibility to stick to their plan every day, or included non‐daily occurring situations in their plans. One participant mentioned for example: *‘I have to. I'm quite strong‐willed in that regard, but my head is a mess, so I'll undoubtedly slip up every now and then, but then I'll be like, ‘No, come on, this is [tapping the paper with her plan several times] what I'm going to do, come on, back to the lesson’.’* (woman, 67, medium education). Participants generally found that their plans fit well with their daily routines (e.g., ‘*My appointments are often in the morning, so I have time in the afternoon to do household chores’*, woman, 52, high education), although some plans were not feasible to implement daily (e.g., improving sleep when working night shifts). Besides strong willingness to implement the generated plan, participants generally expressed the intention to continue using the newly learned strategy beyond the study context, particularly to improve healthy eating and physical activity (e.g., *‘I can see myself doing this on my own […] If I really aim to change something for myself, I could use such a plan myself’*, woman, 20, low education). Overall, participants had positive attitudes towards adopting their generated plan and the strategy more generally in daily life.

##### Positive perceptions of generating implementation intentions

###### Generating implementation intentions is perceived as easy

A key theme identified was that participants generally perceived generating their implementation intention by identifying a cue and response as easy. Reasons for this that stood out most were already actively pursuing one's goal (e.g., *‘It was not difficult, but that is because I am already into it. It plays a role every day […] I am working on it’*, woman, 62, low education), and having insight into one's circumstances, problems and opportunities (*‘Because I know about my circumstances. I know about my problems, and I believe everyone knows the source of their problems and what the solutions are.’* man, 51, high education). Besides, participants generally expressed to feel capable of independently generating new implementation intentions for other behaviours.

###### Generating implementation intentions is pleasant and helps to reflect

Another theme identified was that participants perceived generating their implementation intentions as pleasant. One thing that participants expressed as pleasant, was that generating a plan with the exercise as well as talking about it with the interviewer is a helpful way to brainstorm and reflect on (changing) their health behaviour. For example, one participant mentioned: ‘*I have looked at myself a little. And ‘how am I, where am I, and where should I go?’ There I have looked at myself, so to speak, in the mirror*.’ (man, 62, low education). It was expressed by some participants that (discussing) the exercise helped to stay or become even more motivated to change the health behaviour and work on the plan (e.g., ‘*Now that I have to consciously write this down and discuss it, it is very important to me that I maintain this. […] It helped me to maintain that motivation*’, woman, 62, low education).

##### Social support is an additional need

Another theme was the need for social support, both from experts and peers. Considering expert support, participants valued guidance in formulating implementation intentions, discussing their ideas and brainstorming. Such support could help to come up with new ideas, stay motivated and increase one's determination to implement the plan (*‘Now I am talking to someone about it and then you think, yeah, I should have done that, you know. By yourself, alone, you don't come up with it’*, man, 47, high education; *‘You really helped me […] now you just give me even more strength to do it’*, woman, 42, low education). Besides, participants expressed the need for peer support. Specifically, generating plans together, implementing plans simultaneously, discussing progress, being corrected when one does not stick to their plan, and encouraging each other. One participant mentioned: ‘*I will tell my mother I want to go to bed an hour earlier every night so that she can check me. If I use my phone she could say, ‘you're breaking your rules. You agreed to go to bed earlier’’*, woman, 27, medium education.

#### Independently generating implementation intentions: Process and evaluation of formulated plans (second aim)

Considering the second aim, participants generally requested or received guidance from the interviewer during plan formation. Besides, an evaluation of participants' plans demonstrates that the implementation intentions often lacked the correct if–then structure and concreteness. These themes are described below.

##### Guidance was often requested or provided during generating implementation intentions

A central theme was that participants frequently requested or received guidance from the interviewer. Only few participants completed the task without requesting or receiving substantial support. Requested support often included seeking confirmation, such as whether they had understood the text and the instructions correctly or if their responses were sufficient (e.g., *‘So, if I understand it correctly, I need to mentally prepare myself now, right? By imprinting it in my mind that I will do that in the future?’*, man, 61, medium education). This relates to the theme that some participants experienced textual barriers regarding the written instructions, such as long sentences and many questions or tasks which required considerable effort in terms of reading and writing. Some also mentioned the amount of repetition as challenging, which was reflected in participants requesting guidance regarding combining (or actually repeating) the ‘if’ and ‘then’ elements into a coherent plan (e.g., ‘*What is my plan here? […] But what should I write here then?’* man, 62, low education). Support provided by the interviewer mainly concerned helping participants to understand what was requested from them in each step, and particularly to brainstorm about and specify their answers (i.e., the cue and response). Participants mentioned that they appreciated the received guidance.

##### Formulated plans lack important elements

###### Plans are not formulated in the correct if–then structure

Evaluation of participants' plans revealed that they generally were not formulated in the correct if–then structure. In some final plans, the ‘if’ and/or ‘then’ words were missing (e.g., ‘*After work or activity, relaxing on the couch for a while’*, woman, 64, low education). Besides, the ‘if’ and ‘then’ elements were often not formulated correctly and it was not always specified what the participant actually had to do (e.g., *‘08:00 breakfast, 13:00 fruit, 18:00 dinner’*, man, 61, medium education). Additionally, plans (including the ‘if’ and/or ‘then’ parts) gradually changed across the different steps of the task. Especially when participants were instructed to repeat their plan, they generally filled out something different, such as repeating their goal.

###### Plans are not concrete

Formulated plans generally lacked concreteness, including the ‘if’ situations. Although participants seemed to be able to recognize situations in daily life where they engaged in unhealthy behaviour, they were not able to formulate this situation as a concrete ‘if’ part, as illustrated by the following plan: *‘If times and quality, then being on location where can I eat healthily’* (man, 51, high education). This participant recognized, for example, that he often skipped meals, but did not precisely specify when to eat healthy meals. The lack of concreteness similarly applied to the goal‐directed responses, which sometimes lacked detail (e.g., *‘If I am tired (or not in the mood or have been outside), then I still want to eat healthily’*, woman, 42, low education), or addressed behaviours that the participant currently engaged in and aimed to avoid (e.g., *‘If I get home, I will relax for a bit by watching television and chatting with my wife. Then, I usually drink water and as little as possible cola to get used to that [I] drink less cola’*, man, 62, low education). Plans therefore did not always specify what the participant actually needed to do. Overall, participants could recognize possible situations in daily life and responses that would be appropriate, but these elements were not always formulated in detail for specific, actionable if–then plans.

## DISCUSSION

The present study explored how adults with lower SEP perceive (generating) implementation intentions for health behaviour change and how they independently generate such plans. Using semi‐structured interviews with a think‐aloud methodology, we found that participants were highly motivated to change their health behaviour and generally perceived implementation intentions as helpful and appealing to do so. At the same time, participants frequently requested guidance during plan formation, and their plans often lacked the correct if–then structure and concreteness. Together, these findings indicate that while implementation intentions are positively received among individuals with lower SEP, independently formulating well‐structured plans is challenging.

### Key findings

#### Readiness for behaviour change and using implementation intentions

Participants expressed strong readiness for behaviour change. They had high motivation to improve their health, consistent with research showing that individuals with lower SEP are strongly motivated to live healthily, despite facing barriers to act on these intentions (e.g., Godin et al., 2010; Koivusaari et al., 2025; McCoy et al., 2024). This motivation applied both to general daily health behaviour and to the specific behaviour targeted in their plans. This is important because the effectiveness of implementation intentions depends on a strong goal intention, i.e., the motivation to perform the behaviour specified in the plan (Gollwitzer, 1999; Gollwitzer & Sheeran, 2006; Sheeran et al., 2005).

Importantly, our findings demonstrate that participants were highly motivated not only for the behaviour specified, but also for implementing the plan in daily life. Commitment to the plan itself also influences its effectiveness (Gollwitzer & Oettingen, 2016), but is rarely examined. In line with DeBiasse et al. (2017), participants perceived implementation intentions as helpful for behaviour change. They appreciated the opportunity to reflect on their health behaviour and perceived generating their plan as helpful for consciously determining how to implement behaviour. This strengthened feelings of commitment. Besides willingness to use the generated plan, participants expressed willingness to use implementation intentions for health behaviour change beyond the study context.

Interestingly, participants perceived it helpful that the strategy starts with small steps, provides structure and supports step by step progress towards healthier behaviour. This finding is particularly noteworthy given concerns raised in the introduction that micro‐level interventions like implementation intentions might feel insufficient or demotivating for individuals facing structural challenges. Instead, our finding suggests that the simplicity of the strategy to break behaviour change down into small, concrete and manageable actions may reduce feelings of overwhelm by offering structure, direction and a greater sense that behaviour change is achievable. Such control beliefs may enhance the motivation and willingness to engage with the strategy as well as commitment to the plan among individuals with lower SEP. This interpretation aligns with research showing that lower SEP groups tend to mostly apply information consisting of small, practical, everyday actions they can carry out independently, without requiring substantial time or financial resources (Stormacq et al., 2023). For individuals whose daily lives are already dominated by multiple competing demands, focusing on proximal, attainable actions (Gollwitzer, 1999; Gollwitzer & Oettingen, 2016) may better match their available cognitive resources and circumstances. Thus, even (or maybe particularly) when the problems are so complex among lower SEP populations, focusing on little steps in behaviour change may be practicable and desirable. Altogether, participants' positive perceptions of and willingness to use implementation intentions indicates that ‘simple plans’ may indeed resonate with lower SEP contexts and actually be particularly suitable for adults with lower SEP.

#### Difficulties with independent plan formation

However, the notion ‘simple plans’ also implies that implementation intentions are easy to generate, while this constitutes several cognitive steps. This study extends existing literature by showing *how* individuals with lower SEP construct implementation intentions. Although most participants *perceived* generating their plan as easy, their written plans typically lacked essential structural features. That is, formulations frequently changed across different steps of the task, and the plans often lacked concreteness and the correct if–then format. Poorly specified or vague implementation intentions may fail to support behaviour change (Gollwitzer & Sheeran, 2006), and using the correct if–then format seems crucial for the effectiveness of implementation intentions, possibly because this mirrors the if–then sequence of events in daily life (Bieleke et al., 2021). Our findings align with previous work demonstrating that making simple plans is not that simple (Adriaanse et al., 2011; Adriaanse & Verhoeven, 2018; De Vet et al., 2011). This is also reflected in the finding that guidance was often requested or provided. Participants expressed that they valued this guidance and brainstorming support, consistent with prior acceptability findings (DeBiasse et al., 2017). Thus, in terms of the strategy being simple and easy to use, the notion of ‘simple plans’ may not be applicable for individuals with lower SEP.

### Implications

The findings have several implications for intervention development and future research. First, participants' positive perceptions of using implementation intentions for changing health behaviour highlights potential of the strategy for lower SEP populations, but providing explicit guidance is essential for successful plan formation. Guidance should particularly focus on identifying concrete, recognizable and frequently occurring situational cues and actionable responses and linking them in a correct if–then format concretizing where, when and how to act. Guidance for such better plan formation may include volitional help sheets (e.g., Armitage & Arden, 2010), prescribed plans (e.g., Armitage & Sprigg, 2010) and explaining the ideas behind implementation intentions effectiveness, such as the importance of the if–then elements (being concretely formulated) and sequence. Besides, several participants experienced difficulties with understanding the instructions or formulating their answers, suggesting that intervention developers and researchers should reconsider how they generally approach lower SEP groups and deliver (implementation intentions) interventions (e.g., using alternatives to text‐based delivery). As participants expressed a strong desire for shared reflection and accountability, guidance could be combined with social support components, such as expert‐guided or peer‐based planning. Future research should investigate which type of guidance is most supporting among lower SEP populations.

Second, whether the notion ‘strong effects of simple plans’ applies to individuals with lower SEP remains relatively understudied, particularly for self‐generated implementation intentions. Although many written plans lacked the correct if–then structure and sufficient detail, this does not necessarily imply that participants would be unable to recognize critical cues or enact appropriate responses in real‐life situations. It may be that some participants had more concrete mental representations than were reflected in their formulations. However, because the effectiveness of implementation intentions relies on strong cue‐response links (Gollwitzer & Sheeran, 2006), insufficient specification could limit effectiveness. Future research could therefore examine the effectiveness of self‐generated implementation intentions (versus implementation intentions generated with additional guidance) among individuals with lower SEP.

### Strengths and limitations

Key strengths of this study include its focus on an understudied population in implementation intentions research, specifically addressing acceptability and feasibility prior to effectiveness. By providing insight into participants' perceptions, cognitions and plan formation, this study provides an essential foundation for future research on (the effectiveness of) implementation intentions among lower SEP populations. However, several limitations should be acknowledged. First, the method required a continuous balance for the interviewer between allowing participants to work independently and providing guidance. We aimed to observe how participants generate implementation intentions, but the conversational nature of the interviews occasionally prompted interviewer support. This may have influenced participants' experience and their plans. Second, there may be social desirability bias in evaluating the perceived usefulness and feasibility of the plans, particularly because the interviewer was supportive during the exercise. Third, although participants lived in lower SEP neighbourhoods, individual differences in education, literacy and health experience may have influenced how they understood the instructions and generated implementation intentions. An important avenue for future research is therefore to examine whether the acceptability and independent formation of implementation intentions is influenced by individual differences in education and (health) literacy.

## CONCLUSION

This study provided insight into how adults with lower SEP perceive and generate implementation intentions for health behaviour change. Motivation for health behaviour change was high and the strategy was viewed positively, particularly for its focus on small‐scale behaviours. However, participants needed substantial guidance for plan formation and plans often lacked specificity and the correct if–then structure. These findings highlight that implementation intentions and its notion of ‘simple plans’ can be acceptable and potentially valuable for lower SEP populations, but guided plan formulation is essential for actionable and conceptually correct plans. Only when carefully adapted to individuals' contexts, capabilities and needs, the strategy of using ‘simple plans’ may have its full potential among adults with lower SEP.

## AUTHOR CONTRIBUTIONS

**Loes van den Bekerom:** Conceptualization; data curation; formal analysis; investigation; methodology; project administration; validation; writing – original draft; writing – review and editing. **Laurens C. van Gestel:** Conceptualization; formal analysis; methodology; supervision; validation; writing – review and editing. **Satu Koivusaari:** Formal analysis; investigation; validation; writing – review and editing. **Jet Bussemaker:** Conceptualization; methodology; supervision; validation; writing – review and editing. **Marieke A. Adriaanse:** Conceptualization; methodology; supervision; validation; writing – review and editing.

## FUNDING INFORMATION

No grant was received from any funding agency for conducting this study.

## CONFLICT OF INTEREST STATEMENT

The author(s) declare none.

## ETHICS STATEMENT

The department's science committee positively assessed the current study's quality and feasibility (#WSC‐2024‐41/SP) and the Medical Ethics Review Board of Leiden University Medical Center confirmed that the Medical Research Involving Human Subjects Act (WMO in Dutch) does not apply to the current study according to Dutch standards (#24–3077).

## Supporting information


**Data S1.** Dutch and English Translation Interview Guide.

**Data S2.** Practice and Main Think Aloud Tasks (in Dutch).

**Data S3.** Reflexivity.

## Data Availability

Given the sensitive nature of the data from a small sample size, the data are not publicly available.

## Footnotes

^1^
During the interview, one participant experienced some difficulty with reading and particularly writing, despite sufficient oral proficiency, and received additional support. The participant read all instructions out loud and verbalized their interpretation of the instructions. Only if necessary, the interviewer corrected, repeated, or explained what was written. The interviewer noted the answers of the participant by asking what to write down word for word. This interview therefore took 90 min. This instance highlights that lower SEP groups require additional attention regarding data collection, including careful consideration of accessibility, literacy, and support needs.
