# Supplementary material for: Implementation intentions as an acceptable health behaviour change strategy? Insights from people with lower socio‐economic position in think‐aloud interviews
Source: Br J Health Psychol. 2026 Jul 10;31(3):e70090. doi: 10.1111/bjhp.70090 (PMC13352204; doi:10.1111/bjhp.70090)
Supplement: Supplementary file 1 — Data S1. Dutch and English Translation Interview Guide. [file BJHP-31-0-s002.docx]

**Supplementary File 1 – Dutch and English Translation Interview Guide**

**Interview guide - ‘Think-aloud’ Over Actie Plannen (Dutch, original)**

**Voorafgaand**

- Ruimte klaarzetten (indien op campus: koffie/thee/water/koekje/fruit neerzetten), recorder vast klaarzetten
- Small talk met deelnemer, zodat die zich op diens gemak gaat voelen

**Inleiding: Informatie en IC ondertekenen (5-7 minuten)**

Bedankt voor uw komst. Wat fijn dat wij u mogen interviewen.

[*Voorstellen interviewer*]

Doel: Wij doen onderzoek vanuit het LUMC. Dit onderzoek gaat over gezondheid en gedrag. Er zijn verschillende strategieën die mensen kunnen gebruiken om hun gedrag te veranderen. Met dit onderzoek willen we te weten komen wat inwoners uit Den Haag vinden van één van die strategieën. Dat zijn *actie plannen*. Dat is een duidelijk plan over waar, wanneer en hoe je jouw gedrag kunt uitvoeren. We willen te weten komen wat inwoners uit Den Haag denken over actie plannen, hoe zij de stappen voor het maken van zulke plannen vinden en of deze strategie bij hun dagelijks leven past. Wij vinden uw mening over deze actie plannen erg belangrijk.

Manier: Het interview duurt maximaal 45 minuten. Tijdens dit interview maken we gebruik van ‘*Hardop denken*’ (in het Engels: *Think aloud*). We vragen u tijdens het interview om een *actie plan* te maken en daarbij hardop te denken. Dat betekent dat u alles wat u denkt met mij deelt, door uw gedachten en ideeën hardop uit te spreken. Ook zal ik u vragen stellen. Deze vragen gaan over uzelf, gezondheid en gedrag, en wat u vindt van de stappen en *actie plannen*.

Het gaat om uw gedachten en mening, er zijn geen goede of foute antwoorden. U mag vrij vertellen over uw ervaringen. Als er een vraag is die u niet wilt beantwoorden dan mag dat.

We nemen het interview graag op. Dit helpt ons om de resultaten goed te verwerken. De informatie zal nooit naar u als persoon te herleiden zijn in rapporten over het onderzoek. Vindt u dat goed?

Overige vragen beantwoorden: Heeft u nog vragen over het onderzoek?

- *Informatiebrief geven/doorlopen*
- *Informed consent invullen en ondertekenen > dubbel: 1 voor deelnemer, 1 zelf meenemen*

**Start interview (1 minuut)**

We zullen zo met het interview starten.

Vindt u het goed als dit gesprek vanaf nu wordt opgenomen, zodat we de informatie beter kunnen onthouden en verwerken in ons onderzoek?

- *Geluidsopname starten*

**IJsbreker (5 minuten)**

Voordat we het over gezondheid en gedrag gaan hebben, zou ik u graag eerst wat beter willen leren kennen.

| **Subthema** | **Voorbeeldvragen** |
| --- | --- |
| IJsbreker/beter leren kennen | Kunt u iets over uzelf vertellen?   - Hoe is uw leefsituatie? - Hoe ziet een gemiddelde/typische week er voor u uit? |

**Gezondheid (5 minuten)**

Ik zou het nu graag met u over gezondheid willen hebben.

| **Subthema** | **Voorbeeldvragen** |
| --- | --- |
| Gezondheid | Welke rol speelt gezondheid voor u in uw leven?   - Waarom? - Bent u ook bezig met het verbeteren van uw gezondheid?   - Zo ja, op welke manier?   - Welke plek neemt dat in uw leven in? |

**Oefen opdracht (7 minuten)**

Voordat we het gaan hebben over de strategie die u kan helpen om gedrag te veranderen, zou ik graag een oefenopdracht met u willen doen om het hardop denken met u te oefenen. Ik geef u zo een opdracht over het plannen van iets uit het dagelijks leven. Dit gaat dus niet over gezondheid, maar even over iets totaal anders. Deze opdracht is alleen bedoeld om het hardop denken te oefenen. Straks zullen we het weer over gezondheid hebben.

- *Instructie voor hardop nadenken: “Zou u de informatie en instructies hardop willen lezen, en bij het uitvoeren van de instructies hardop willen nadenken? Met ‘hardop nadenken’ bedoel ik dat u alles wat er door uw gedachten heen gaat hardop uitspreekt tijdens het lezen en beantwoorden van de vragen.*

*Alles wat er door uw hoofd gaat tijdens het lezen en beantwoorden van de vragen is waardevol. Zou u daarom alles wat u leest en denkt hardop willen zeggen? Dit is bijvoorbeeld hardop vertellen hoe u een vraag of opdracht begrijpt, wat u denkt dat er gevraagd wordt en wat u vindt van uw mogelijke antwoord. Als een vraag of opdracht moeilijk of onduidelijk voor u is, mag u dat uiteraard ook zeggen.*

*Ik zou u willen vragen om de hele tijd hardop te denken en te praten. Er zijn geen goede of foute gedachten of antwoorden. Doe alsof u alleen in deze kamer bent en tegen uzelf aan het praten bent.*

*Ik zal stil zijn terwijl u de oefenopdracht uitvoert en hardop denkt. Zodra u een tijdje stil bent, zal ik u vragen om verder te gaan met het hardop nadenken. Als u ergens geen antwoord op weet, mag u ook verder gaan met het volgende onderdeel.”*

- *De oefenopdracht geven (zie apart bestand)*

Bedankt voor het uitvoeren van deze oefen opdracht en het hardop nadenken.

- Hoe vond u het om hardop na te denken tijdens deze oefen opdracht?

Zo meteen zullen we verder gaan met het onderzoek over gezondheid. Hierbij zult u weer een opdracht krijgen.

- Heeft u op dit moment nog vragen?

**Onderzoek – Gedrag (7 minuten)**

Het oefen-onderdeel zit erop. We zullen het vanaf nu over gedrag en actie plannen hebben. Eerst moeten we bedenken voor welk gezonde gedrag u een actie plan zou willen maken.

Gedrag: Veel mensen willen gezond zijn en gezonde keuzes maken, zoals voldoende bewegen, gezond eten en voldoende slapen. U zult zo meteen een plan maken om dat gedrag uit te voeren. Welk gedrag zou u willen veranderen in uw dagelijks leven? *(Indien deelnemer er niet direct zelf opkomt, zeggen dat er gedacht kan worden aan … (Bijv. eten (eten van groente/eten van fruit), bewegen, slaap etc. Innemen van medicijnen, gezondheidscheck)*).

- *Gedrag vaststellen*

| **Sub thema** | **Voorbeeldvragen** |
| --- | --- |
| **Gedrag** | |
| Motivatie | Hoe gemotiveerd bent u om uw [gekozen gedag] te veranderen?   - Waarom wilt u dit gedrag veranderen? - Hoe belangrijk vindt u het om dit gedrag te veranderen? |

**Actie plan opdracht (10 minuten)**

Ik zal u zo een oefening geven om een actie plan te maken. Dit plan kan u helpen om uw [*gekozen gedrag*] te veranderen.

- *Instructie voor hardop nadenken: “Ik geef u nu de instructie voor het maken van een actie plan. We hebben ons best gedaan om de instructies zo duidelijk mogelijk te maken. Zou u de instructies hardop willen lezen en deze stap-voor-stap willen uitvoeren. Blijf terwijl u de opdracht uitvoert de hele tijd hardop praten en probeer alles te zeggen wat er door u heen gaat. Alles wat u denkt tijdens en na het lezen en beantwoorden van de vragen is waardevol voor ons. Er zijn geen goede of foute gedachten of antwoorden. Doe alsof u alleen in deze kamer bent en tegen uzelf aan het praten bent. Ik zal stil weer stil zijn terwijl u de opdracht uitvoert en hardop denkt.”*
- *Planning opdracht geven (apart bestand)*
- *Eventueel aanmoedigende opmerkingen maken indien deelnemer vastloopt.*
- *Als deelnemer voor 10-15 seconden stil is: “Zou u alstublieft hardop willen blijven nadenken? Alles dat door uw gedachten gaat tijdens het lezen en beantwoorden van de vragen is waardevol.”*
- *Als deelnemer langer dan 10-15 seconden stil blijft (of vaak 10-15 seconden): “Vindt u het fijn als ik de instructies voor het hardop nadenken nog eens voor u voorlees?”*
- *Als deelnemer vragen heeft over de inhoud/begrip daarvan: “Hoe begrijpt u de vraag/opdracht? Kunt u die in eigen woorden navertellen?”*
- *Als deelnemer herhaaldelijk dezelfde vraag stelt: “Deze vraag gaat over..”/”Deze instructie vraagt van u om…”*

Bedankt voor het afronden van deze opdracht.

**Aanvullende vragen (15 minuten)**

Ik zou u nu graag vragen naar uw ervaringen met het uitvoeren van deze opdracht, en wat u vindt van actie plannen.

Specifieke plan: Eerst zou ik het graag met u willen hebben over het plan dat u net zelf gemaakt heeft.

| **Sub thema** | **Voorbeeldvragen** |
| --- | --- |
| **Uitvoeren van de opdracht** | |
| Algemeen | Hoe vond u het om een actieplan te maken met de stappen?   - Waarom vindt u dat? - Wat vond u van de instructies?   - Wat vond u fijn in het volgen van de instructies? / Wat zou u zo willen houden in de instructies?   - Wat vond u niet fijn in het volgen van de instructies? / Wat zou u anders willen in de instructies?   Hoe makkelijk of moeilijk was het voor u om een plan te vormen? (plus focus op als / dan)   - Hoe was het voor u om het ‘als’ deel van het plan te bepalen? Waarom?   - Waarom heeft u voor dit moment gekozen? - Hoe was het voor u om het ‘dan’ deel van het plan te bepalen?   - Waarom heeft u voor dit gedrag gekozen? - Hoe was het voor u om het voor te stellen? |
| **Als-dan plan** | |
| Effectiviteit actieplan | Ziet u het gebruik van dit plan zitten om uw gedrag aan te passen?   - Waarom wel/niet? - In hoeverre denkt u dat dit plan u zouden kunnen helpen om het [gekozen gedrag] te veranderen? - Denkt u dat dit plan u zou kunnen helpen om gezonder te kunnen leven? - Heeft dit plan ervoor gezorgd dat u anders kijkt naar [gekozen gedrag]? Op welke manier? - Waar helpt het plan u mee? - Waar helpt het plan u niet mee? |
| **Plan in dagelijks leven** | |
| Bruikbaarheid | In hoeverre denkt u dat u dit plan kunt uitvoeren?   - Waarom (wel/niet)?   Hoe past dit plan bij uw dagelijkse bezigheden?   - Waarom past dit wel/niet goed?   Er zijn in het leven natuurlijk altijd andere dingen die meer prioriteit vragen/belangrijker zijn. In hoeverre sluit het plan daarop aan?   - Waarom?   Denkt u dat u dit plan zou kunnen volhouden van dag tot dag?   - Waarom zou u dit kunnen lukken? - Wat belemmert u om dit te kunnen doen? |

Toepassing op meer situaties en gedragingen: Nu zou ik het graag met u willen hebben over het gebruiken van dit soort plannen voor meer situaties en gedrag.

| **Sub thema** | **Voorbeeldvragen** |
| --- | --- |
| **Begrip actieplannen** | |
| Bedoeling actieplan | Als u denkt aan het gebruik van dit soort plannen voor meer situaties en ander gedrag, kunt u in eigen woorden uitleggen hoe u een actie plan zou kunnen maken?   - Kunt u uitleggen uit welke onderdelen een actie plan bestaat? - Kunt u een voorbeeld geven hoe een actie plan gemaakt wordt? |
| Autonomie gehele strategie | Hoe is het voor u om zelfstandig met deze strategie, het maken van actie plannen, aan de slag te gaan in het dagelijks leven?   - Waarom is dat?   Denkt u dat u deze strategie, het maken van actie plannen, zou kunnen gebruiken voor ander gezond gedrag?   - Welk gedrag? Waarom dat gedrag?   Zou u zelfstandig deze strategie toe kunnen passen, zonder hulp?  Denkt u dat het gebruik van deze strategie behulpzaam is voor het veranderen van uw gezonde gedrag? |
| **Afsluitend** | |
|  | Wat vindt u het belangrijkste van ons gesprek vandaag?   - Hebben we nog iets belangrijks gemist?/Wilt u zelf nog iets naar voren brengen?   Heeft u nog vragen of opmerkingen over dit gesprek of het onderzoek? |

**Afronding (7 minuten, inclusief vragenlijst invullen)**

Hartstikke bedankt voor uw tijd en inzet tijdens dit interview. Zou u nog een aantal korte vragen over uzelf willen invullen? Aan het eind van deze vragen kunt u aangeven of u op de hoogte gebracht wilt worden van de resultaten.

- *Vragenlijstje laten invullen (5 minuten), en bedanken voor deelname.*
- *Opname stoppen.*
- *Beloning (cadeaubon) geven.*
- *Foto laten maken van formulier (zelf formulier actieplan meenemen).*

**Interview guide - ‘Think-aloud’ on Implementation intentions (English, translation)**

**Preparations**

- Prepare the room (if at university: provide coffee/tea/water/biscuit/fruit), set up the voice recorder in advance.
- Engage in small talk with the participant so they feel at ease.

**Introduction: Information and Informed Consent (5-7 minutes)**

Thank you for coming. We are very pleased to interview you.

[*Interviewer introduces herself*]

Purpose: We are conducting research at LUMC. This study focuses on health and behavior. There are various strategies people can use to change their behavior. With this study, we want to understand what residents of The Hague think about one of these strategies. That are If-Then plans. An If-Then plan is a clear plan about where, when, and how you will perform a behavior. We would like to learn what residents of The Hague think about If-Then plans, how they experience the steps involved in creating such plans, and whether this strategy fits their daily lives. Your opinion about such If-Then plans is very important to us.

Procedure: The interview will take a maximum of 45 minutes. During the interview we will use a ‘***Think-aloud’*** method. During the interview, we will ask you to create an If-Then plan and to think aloud while doing so. This means that you share everything you are thinking with me by expressing your thoughts and ideas out loud. I will also ask you questions. These questions are about yourself, health and behavior, and what you think about the steps and If-Then plans.

This interview is about your thoughts and opinions. There are no right or wrong answers. You are free to talk about your experiences. If there is a question you prefer not to answer, that is perfectly fine.

We would like to audio-record the interview. This helps us to process the results accurately. The information will never be traceable to you as a person in reports about the study. Is that okay with you?

Remaining questions: Do you have any questions about the study?

- *Provide the information letter and informed consent and discuss it.*
- *Complete and sign the informed consent form (two copies: 1 for the participant, 1 for the researcher)*

**Start of the interview (1 minute)**

We will now begin the interview.

Do you give permission that this conversation is recorded from now on, so that we can better remember and process the information for our research?

- *Start audio recording*

**Icebreaker (5 minutes)**

Before we will talk about health and health behavior, I would like to get to know you a bit better.

| **Subtheme** | **Example questions** |
| --- | --- |
| Icebreaker/getting to know the participant better | Could you tell me something about yourself?   - What is your living situation like? - What does an average/typical week look like for you? |

**Health (5 minutes)**

I would now like to talk with you about health.

| **Subtheme** | **Example questions** |
| --- | --- |
| Health | What role does health play in your life?   - Why? - Are you working on improving your health?   - If yes, in what way?   - How important if this in your daily life? |

**Practice task (7 minutes)**

Before we will talk about the strategy that can help to change behavior, I would like to do a practice task with you to get used to thinking aloud. I will give you an assignment about planning something in everyday life. This is not about health, but about something completely different. This task is only meant to practice thinking aloud. Afterwards, we will return to the topic of health.

- *Instruction for thinking aloud: “Could you please read the information and instructions out loud, and think aloud while carrying out the instructions? By ‘thinking aloud,’ I mean that you say everything that goes through your mind out loud while reading and answering the questions.*

*Everything that goes through your mind while reading and answering the questions is valuable. Could you therefore please say out loud everything you read and think? This includes, for example, tell out loud how you understand a question or task, what you think is being asked, and what you think about your possible answer. If a question or task is difficult or unclear, you can say that as well of course.*

*I would like to ask you to think and talk aloud the entire time. There are no right or wrong thoughts or answers. I would like to ask you to pretend that you are alone in this room and talking to yourself.*

*I will remain quiet while you complete the practice task and think aloud. If you are quiet for a while, I will ask you to continue thinking aloud. If you do not know the answer to something, you may move on to the next part.”*

- *Provide the practice task (see separate document)*

Thank you for completing the practice task and thinking aloud.

- How did you experience thinking aloud during this practice task?

In a moment, we will continue with the study about health. You will then receive another task while thinking aloud.

- Do you have any questions at this point?

**Behavior (7 minutes)**

The practice part is now finished. From this point on, we will talk about behavior and If-Then plans. First, we need to decide which health behavior you would like to create an action plan for.

Behavior: Many people want to be healthy and make healthy choices, such as being physically active, eating healthily, and getting enough sleep. In a moment, you will create a plan to perform such a behavior. Which behavior would you like to change in your daily life? (*If the participant does not come up with one, suggest examples such as diet (eating vegetables/fruit), physical activity, sleep, taking medication, or attending health check-ups.*)

- *Determine health behavior*

| **Subtheme** | **Example questions** |
| --- | --- |
| **Behavior** | |
| Motivation | How motivated are you to change your [chosen behavior]?   - Why do you want to change this behavior? - How important is it to you to change this behavior? |

**Main task If-Then plan (10 minutes)**

I will now give you an exercise to create an If-Then plan. This plan can help you to change your [chosen behavior].

- *Instruction for thinking aloud: “*I will now give you the instructions for creating an If-Then plan. We have done our best to make the instructions as clear as possible. Could you please read the instructions out loud and carry them out step by step? While doing so, please keep talking aloud and try to say everything that goes through your mind. Everything you think during and after reading and answering the questions is valuable to us. There are no right or wrong thoughts or answers. Please act as if you are alone in this room and talking to yourself. I will again remain quiet while you complete the task and think aloud.”
- *Provide the main planning task (separate document)*
- *If necessary, provide encouraging prompts if the participants gets stuck, such as:*
- *If the participant is silent for 10–15 seconds: “Could you please continue thinking aloud? Everything that goes through your mind while reading and answering the questions is valuable.”*
- *If the participant remains silent for longer than 10–15 seconds (or repeatedly 10-15 seconds): “Would it be helpful if I read the instructions for thinking aloud again?”*
- *If the participant has questions about the content or understanding: “How do you understand the question/task? Could you explain it in your own words?”*
- *If the participant repeatedly asks the same question: “This question is about…” / “This instruction asks you to…”*

Thank you for completing this task.

**Additional questions (15 minutes)**

I now would like to ask you about your experiences with performing this task and what you think about If-Then plans.

Specific plan: First, I would like to talk about the plan that you just created.

| **Subtheme** | **Example questions** |
| --- | --- |
| **Performing the task** | |
| General | How did you experience creating an action plan using these steps?   - Why do you feel that way? - What did you think of the instructions?   - What did you like about following the instructions? / What would you keep the same?   - What did you not like about following the instructions? / What would you like to change?   How easy or difficult was it for you to form a plan? (with specific focus on the if/then components)   - How was it for you to determine the ‘if’ part of the plan? Why?   - Why did you choose this moment? - How was it for you to determine the ‘then’ part of the plan?   - Why did you choose this behavior? - How was it for you to imagine the plan? |
| **If-Then plan** | |
| Effectiveness If-Then plan | Do you see yourself using this plan to change your behavior?   - Why or why not? - To what extent do you think this plan could help you change your [chosen behavior]? - Do you think this plan could help you live a healthier life? - Did this plan make you look differently at your [chosen behavior]? In what way? - What does the plan help you with? - What does the plan not help you with? |
| **Plan in daily life** | |
| Usability | To what extent do you think you could carry out this plan?   - Why (not)?   How does this plan fit into your daily activities?   - Why does it fit (not) well?   In everyday life, there are always other things that take priority or are more important. To what extent does the plan take this into account?   - Why?   Do you think you could maintain this plan from day to day?   - Why do you think this would work for you? - What might hinder you from doing this? |

Application to other situations and behaviors: Now I would like to talk with you about using such types of plans for other situations and behavior.

| **Subtheme** | **Example questions** |
| --- | --- |
| **Understanding If-Then plans** | |
| Purpose If-Then plans | When you think about using this type of plans for other situations and behaviors, could you explain in your own words how you would create an If-Then plan?   - Can you explain which components an If-Then plan consists of? - Can you give an example of how an If-Then plan is created? |
| Autonomy strategy | How do you feel about independently using this strategy—creating If-Then plans—in your daily life?   - Why do you feel that way?   Do you think you could use this strategy—creating If-Then plans—for other health behaviors?   - Which behavior(s)? Why those behavior(s)?   Do you think you could apply this strategy independently, without support?  Do you think using this strategy is helpful for changing your health behavior? |
| **Final questions** | |
|  | What do you consider the most important thing of our conversation today?   - Is there anything important we may have missed?/Is there anything else you would like to add?   Do you have any questions or comments about this interview or the study? |

**Wrap-up (7 minutes, including completing short demographic survey)**

Thank you very much for your time and effort during this interview. Would you be willing to fill out a short survey with questions about yourself? At the end of this survey, you could indicate whether you would like to receive the results of this study.

- *Provide survey and let participants complete it (5 minutes), and thank them for participating.*
- *Stop voice recorder.*
- *Provide reward (gift card).*
- *Take a photo of the form(s)/have a photo taken of the form(s). Most important for research: main task for If-Then plans.*
