# Supplementary material for: Implementation intentions as an acceptable health behaviour change strategy? Insights from people with lower socio‐economic position in think‐aloud interviews
Source: Br J Health Psychol. 2026 Jul 10;31(3):e70090. doi: 10.1111/bjhp.70090 (PMC13352204; doi:10.1111/bjhp.70090)
Supplement: Supplementary file 2 — Data S2. Practice and Main Think Aloud Tasks (in Dutch). [file BJHP-31-0-s003.docx]

**Supplementary File 2 – Practice and Main Think Aloud Tasks (in Dutch)**

***Oefen opdracht***

*Denk alstublieft aan de volgende situatie:*

*Stel u voor dat u een mooie foto van uw familie heeft gemaakt. U wilt deze aan de muur ophangen. U heeft niet de materialen in huis om dit te kunnen doen.*

*Bedenk wat u allemaal moet doen om de foto op te hangen.*

*Schrijf hieronder op wat u allemaal gaat doen. Beschrijf dit zo precies mogelijk.*

|  |
| --- |

U bent nu klaar met de oefening!

***Oefening om uw gedrag te plannen***

***Voornemen***

Welk gezond gedrag heeft u net gekozen om te veranderen? Schrijf uw voornemen voor het gedrag op:


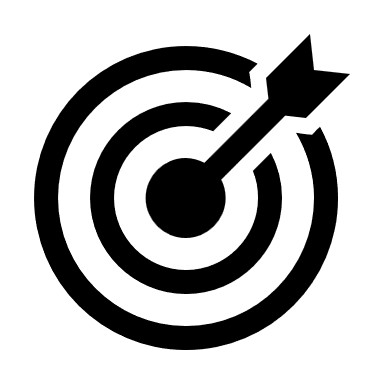


| **Ik ga:**  *Voorbeeld: “Ik ga meer fruit eten”* |
| --- |

***Plan maken***

Om uw goede voornemen uit te voeren, kan een duidelijk *actie plan* helpen. Dit is een Als-Dan plan. Een voorbeeld van een Als-Dan plan voor iemand die meer fruit wilt gaan eten, is: “Als ik thuiskom van werk, dan pak ik een appel van de fruitschaal”. Dit is een voorbeeld en hoeft dus niet geschikt te zijn voor uw situatie. In drie stappen gaat u nu uw eigen *actie plan* maken.

**Stap 1: Als**

Bij deze stap is het belangrijk dat u goed nadenkt over de situatie waarin u uw gedrag kan uitvoeren. Die situatie kan van alles zijn: een *tijd van de dag*, een *plaats* of *activiteit*, een *gevoel* of *gezelschap*.

Wat is een goed moment om het gedrag uit te voeren? Beschrijf die situatie zo precies mogelijk.


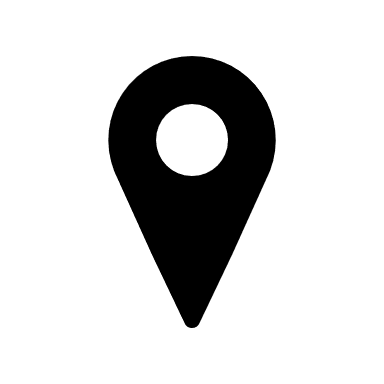


| **(1) Als:**  *Voorbeeld: “Als ik thuiskom van werk”* |
| --- |

**Stap 2: Dan**

Bij deze stap is het belangrijk dat u goed nadenkt over welk gedrag u precies zou kunnen uitvoeren in de door u gekozen situatie. Het gedrag kun je omschrijven met: *hoeveel/hoe vaak*, en welk *gedrag precies*.

Welk gedrag kan ik in de situatie van stap 1 uitvoeren? Beschrijf dat gedrag zo precies mogelijk.

| **(2) Dan:**  *Voorbeeld: “Dan pak ik een appel van de fruitschaal”* |
| --- |

**
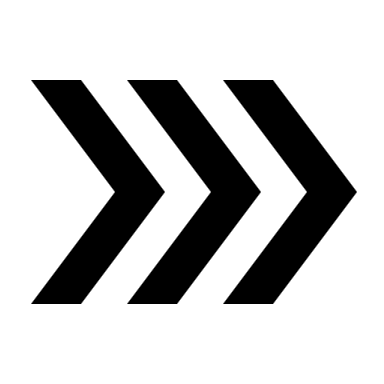
**

**Stap 3: Als-Dan combineren**

U hebt nu een goed moment (Als) en het gedrag (Dan) opgeschreven. Nu kunt u het *actie plan* opschrijven. Dat kan door stap 1 en 2 te combineren.

Mijn *actie plan*:

| 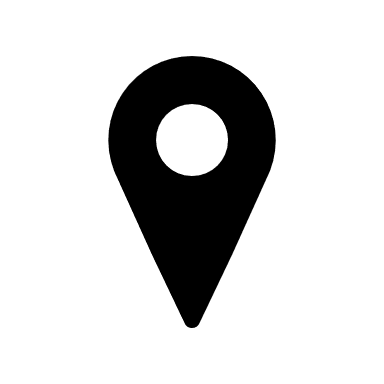**(1) Als:**  **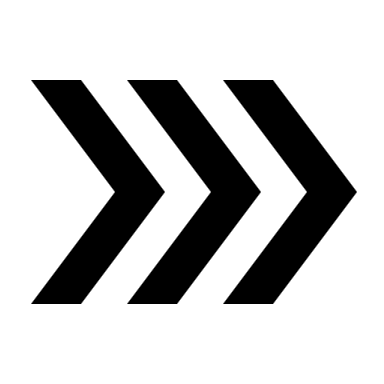**  **(2) Dan:**  *Voorbeeld: “Als ik thuiskom van werk, dan pak ik een appel van de fruitschaal”* |
| --- |

***Herhalen***

Het helpt om u in te beelden dat u het *actie plan* uitvoert. Neem even de tijd om echt voor u te zien dat u het *actie plan* uitvoert. Probeer stap voor stap voor u te zien hoe u het gedrag uitvoert in die situatie. Als u het fijn vindt, kunt u even uw ogen sluiten.

Heeft u uw *actie plan* in gedachten uitgevoerd? Schrijf dan nu uw *actie plan* nog een keer op:

| *Voorbeeld: “Als ik thuiskom van werk, dan pak ik een appel van de fruitschaal”* |
| --- |

U bent nu klaar met de oefening!
