# Supplementary material for: Implementation intentions as an acceptable health behaviour change strategy? Insights from people with lower socio‐economic position in think‐aloud interviews
Source: Br J Health Psychol. 2026 Jul 10;31(3):e70090. doi: 10.1111/bjhp.70090 (PMC13352204; doi:10.1111/bjhp.70090)
Supplement: Supplementary file 3 — Data S3. Reflexivity. [file BJHP-31-0-s001.docx]

**Supplementary File 3 – Reflexivity**

The research team approached this study from a health psychology perspective, with a specific focus on self-regulation processes and health behavior change in socio-economically disadvantaged contexts. Consistent with qualitative approaches that view knowledge as co-constructed between researchers, participants and the research context, reflexivity was applied during study design, recruitment, data collection, analysis, and interpretation to critically consider how researchers’ backgrounds, assumptions, experiences, theoretical perspectives, and interactions with participants could shape both the data generation and interpretation process (Braun & Clarke, 2020).

The first author (LvdB), who recruited participants, and conducted and coded all interviews, is a White Dutch cisgender woman in her 30s. All other researchers are White Dutch or Finnish, including three cisgender women and one cisgender man. The research team includes four social and health psychologists and one researcher with a background in political sciences and governance. All researchers study socio-economic health inequalities, and examine what matters to people with lower socio-economic position (SEP) and which interventions work for this group specifically. These disciplinary and theoretical orientations shaped how the research problem was conceptualized, how data collection was designed (including the think-aloud method), and what aspects of participants’ accounts were attended to during analysis and interpretation. For example, the team’s familiarity with health psychology, self-regulation theories, and implementation intention research sensitized the team to issues such as the specificity of implementation intentions, the identification of cues and responses, perceived feasibility, and participants’ perceptions, motivation and perceived control regarding changing their behavior using implementation intentions.

The research team also reflected on how socio-economic differences between participants and them shaped the research encounter. All researchers were aware that participants’ lived experiences and circumstances can vary extensively from theirs, and that these differences could influence communication, interpretation, and meaning-making during the interviews. LvdB consciously paid attention to interpersonal dynamics, used accessible language, spent time in the community centers and food aid stores prior to recruitment, and engaged in low-key conversations unrelated to the research project before introducing the study. These efforts likely shaped what participants chose to disclose, how comfortable they felt expressing uncertainties during the think-aloud task, and the kinds of meanings that were jointly explored during interviews. During the think-aloud procedure, LvdB balanced between encouraging participants to independently formulate implementation intentions and offering clarification or support when participants appeared to struggle. Importantly, this was not only based on explicit requests for help, but also on non-verbal and interactional cues, such as prolonged silences, hesitations, or questioning facial expressions directed toward LvdB. Such observations were documented in field notes and interpreted as indications that participants experienced difficulties with the task of generating an implementation intention. These interactions and interpretations were considered part of the co-constructed data generation process and were reflected upon during analysis. The co-authors had no direct contact with the participants.

Throughout the study, researchers reflected on how their own perspectives and interest in behavior change interventions and implementation intentions may have influenced interpretations of the participants’ responses and independent plan formation. For example, the research team discussed how participants’ difficulties with independently generating implementation intentions could be interpreted in relation to important aspects of successful plan formation (e.g., the if-then formulation, cognitive processes in identifying the critical cue and an appropriate goal-directed response), cognitive challenges and abstract planning tasks, and/or participants’ socio-economic background (e.g., literacy). Reflexivity was further supported through iterative data collection (e.g., adjusting the instructions of the main think-aloud task, the interview guide or code scheme), coding, interpretation, ongoing discussions among the research team and reflections of coding decisions (LvdB, SK and LvG) and emerging interpretations and themes.
